# Are Species Coexistence Areas a Good Option for Conservation Management? Applications from Fine Scale Modelling in Two Steppe Birds

**DOI:** 10.1371/journal.pone.0087847

**Published:** 2014-01-31

**Authors:** Rocío Tarjuelo, Manuel B. Morales, Juan Traba, M. Paula Delgado

**Affiliations:** 1 Terrestrial Ecology Group (TEG), Department of Ecology, Universidad Autónoma de Madrid, Madrid, Spain; University of Lleida, Spain

## Abstract

Biotic interactions and land uses have been proposed as factors that determine the distribution of the species at local scale. The presence of heterospecifics may modify the habitat selection pattern of the individuals and this may have important implications for the design of effective conservation strategies. However, conservation proposals are often focused on a single flagship or umbrella species taken as representative of an entire assemblage requirements. Our aim is to identify and evaluate the role of coexistence areas at local scale as conservation tools, by using distribution data of two endangered birds, the Little Bustard and the Great Bustard. Presence-only based suitability models for each species were built with MaxEnt using variables of substrate type and topography. Probability maps of habitat suitability for each species were combined to generate a map in which coexistence and exclusive use areas were delimitated. Probabilities of suitable habitat for each species inside coexistence and exclusive areas were compared. As expected, habitat requirements of Little and Great Bustards differed. Coexistence areas presented lower probabilities of habitat suitability than exclusive use ones. We conclude that differences in species' habitat preferences can hinder the efficiency of protected areas with multi-species conservation purposes. Our results highlight the importance of taking into account the role of biotic interactions when designing conservation measurements.

## Introduction

The distribution of species is the result of evolutionary, ecological or anthropogenic processes that operate at different spatial and temporal scales [1]–[4]. Climate has been described to play a major role in shaping the distribution of the species at continental and regional scales, while biotic interactions are generally considered secondary [5], [6] but see [7], [8]. Land use and biotic interactions become relevant at local scale, at which they exert a major effect in the configuration of population and community dynamics [9], [10].

The presence of heterospecifics has been proposed as a factor influencing the habitat use of organisms at local scale [11]. Coexistence of sympatric species may be mediated by the segregation of shared resources [12], for example, the differentiation of habitat preferences at landscape or at microhabitat scale [13], or changes in a species' behavioural and food resource-use patterns [14]. Thus, direct or indirect interactions may condition the occurrence of heterospecifics in space and further, the fitness of the individuals [14]. This may be especially relevant for species subject to conservation efforts, since potential changes in habitat use patterns due to biotic interactions may affect their distribution at local scale [9], [15], [16].

In recent years, conservation issues from both theoretical and applied approaches have been increasingly addressed by the use of species distribution models (SDMs) [4], [17]–[20]. SDMs use species occurrence records to infer the environmental conditions under which a species exists in a particular context and further, they allow to predict potential geographic distribution areas. Despite the potential importance of biotic interactions in determining the spatial distribution patterns of species at fine scale, SDM studies usually focus on single, often keystone or umbrella species [4], [21]. However, the efficacy of umbrella and flagship species as conservation tools for protecting other species in the community has been questioned [22], [23], and several studies have highlighted the importance of considering more than one species in designing successful conservation measures [24], [25]. Conservation efforts should be directed towards groups of interacting species, focusing on areas that encompass species assemblages despite the lack of information about interaction networks [26].

In this context, the present study focuses in two steppe bird species which coexist in many areas of their distribution range: The Little Bustard (*Tetrax tetrax*) and the Great Bustard (*Otis tarda*). The two species are of high conservation concern since both are globally endangered and classified as near threatened and vulnerable respectively [27]. Nowadays, Spain holds more than half of their global population [28], [29], being agricultural intensification and the increase of infrastructure development two major causes of population decline and distribution shrink [28], [30]. The Little Bustard is a medium sized steppe bird, which prefers heterogeneous agricultural landscapes that maintain a high proportion of fallows and short natural vegetation [31], [32]. The Great Bustard is one of the heaviest flying birds and shows preference for stubbles, leguminous crops and fallows, although its habitat selection pattern changes seasonally and differs greatly between regions [33], [34]. Both species avoid man-made structures, such as buildings, roads and tracks [33], [35], [36]. To the best of our knowledge, no studies have been conducted at local scale on the Little and the Great Bustards together in order to integrate their habitat preferences for the management of areas in which both species coexist.

Therefore, the aim of this study is to provide useful guidelines for the conservation of these two sympatric species with different habitat preferences through the identification and environmental characterization of coexistence areas at landscape scale. The delimitation of areas in which species are more likely to coexist might help focusing management efforts on the benefit of both species. We discuss the implications of using coexistence areas to conserve species that differ in their habitat preferences.

## Methods

### Ethics Statement

The present study did not required the capture or handling of protected or endangered animals. All data about species' locations were collected by observation at distance using binoculars. The described field studies were carried out on privately-owned farms with the permission of farmers.

### Study sites

The study was carried out in two localities of central Spain, Campo Real sited in Madrid province (40°19′N, 3°18′W. 1 145 ha) and Calatrava, in Ciudad Real province (38°54′N, 3°53′W. 9 016 ha). Both regions are under a Mediterranean climate with annual mean precipitations around 550 mm. These sites are flat to slightly undulated, encompassing mosaics of different agrarian substrates. Extensive dry cereal croplands and ploughed lands make up more than 50% of the surface, with a varying cover of fallows of different ages, leguminous crops and interspersed patches of olive groves, vineyards and fruit tree orchards. Pasturelands and scrublands are also present but in a low percentage.

### Little and Great Bustard data

Little and Great Bustard data were collected between March–April 2008 and 2009 in Calatrava and April–May 2011 and 2012 in Campo Real, during the period of reproductive activity of both species [37]. Surveys were made by car routes throughout the available roads and tracks that cover the entire study site, stopping at every 500 m to ensure the record of all individuals, which were geo-referenced. Each study site was surveyed simultaneously by two car-teams, each composed by two experienced observers and covering a half of the study area, in order to fully cover the study site in a single bustard daily activity period. Surveys were made within the first three hours after daybreak and the three hours before sunset since this is the moment of highest activity, and thus individuals are easier to detect [37]. Only Little Bustard males were considered in this study since females are very difficult to observe due to their secretive behaviour. The detectability of Little Bustard males and Great Bustard males and females were almost complete since the vegetation height is relatively low at this time of the year. In addition, Little Bustard males were also detected acoustically. The Great Bustard presents a lek mating system in which individuals tend to aggregate around conspecifics [30], [38]. Thus, Great Bustard individuals observed in the same flock were considered as a single occurrence record in subsequent analyses in order to avoid the potential effects that conspecific clustering could have in the modelling process.

### Environmental predictors

**Figure 1:**
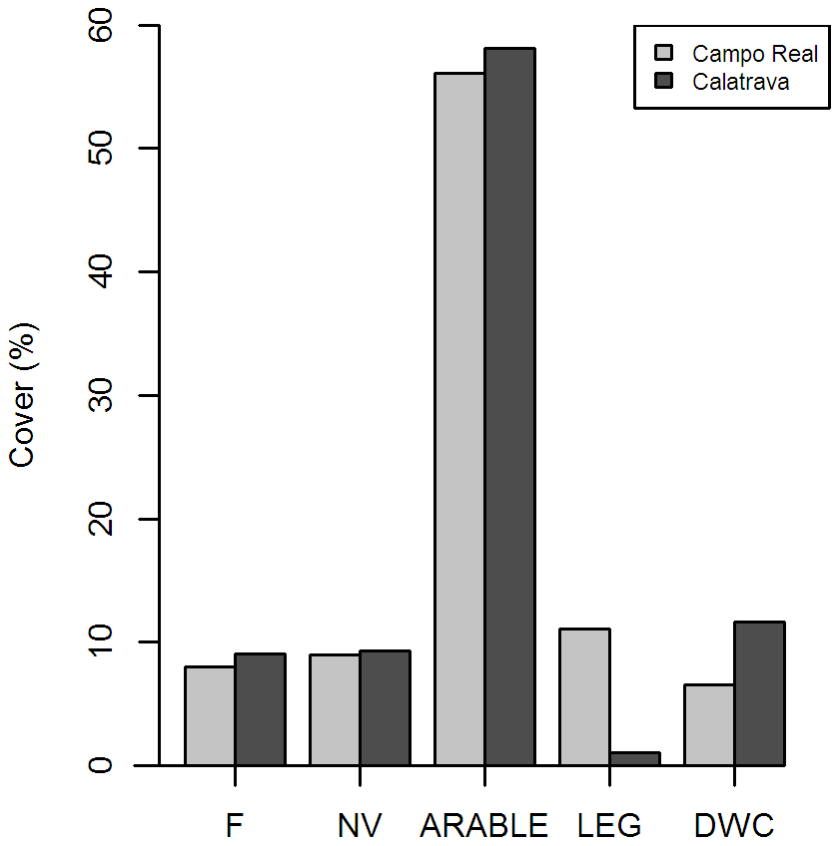
Land use cover in the study sites. Cover percentage of the land uses considered for Maxent modelling in 2011 in Campo Real and 2008 in Calatrava (F: short term fallows, NV: natural vegetation encompassing long term fallows and low height scrubs; Arable: cereal fields and ploughed lands; LEG: leguminous crops; DWC: dry woody cultures).

We used as environmental predictors variables related to substrate types and topography according to existing ecological knowledge on the species [32]–[35]. All the environmental variables were rasterized for model calibration, considering a cell size of 50×50 m. Land-use variables were extracted from land-use maps elaborated from field surveys in each study site and year. Fields on land-use maps were classified regarding their potential to affect the presence of Little and Great Bustards. Thus, agricultural habitat types were: 1) arable lands, including cereal crops and ploughed lands, 2) leguminous crops, which are important for both Little and Great Bustards [33], [39], [40], 3) young fallows (hereafter referred to as fallows), 4) fallows of more than two years and low height scrublands (hereafter called natural vegetation), 5) dry woody cultures which include olive groves and vineyards, 6) others, which comprises urban substrates, fruit tree orchards and forest (Fig. 1). Land-use rasters reflect the proportion of the corresponding land use inside each cell. Land-use proportion was calculated taking into account all land-use categories, so that the sum of all of them was 1 for each cell. As it is highly recommended to reduce the number of variables for model calibration [41], the variable Others was not considered for the analysis since both species avoid the agricultural substrates enclosed in this category [37], [40].

A topography position index (TPI) was also calculated from the digital elevation model, constructed from maps of five meter elevation contour lines. This index was calculated as the elevation value of each cell minus the mean elevation of the neighbouring cells within a particular radius. In this study, a radius of 250 m was selected according to the biological characteristics of the species, given their size and their lek mating system [30], [42]. Therefore, it classifies each cell regarding the elevation of the neighbour cells, reflecting how visible a particular location is. From a behavioural point of view, the selection of areas according to their visibility could result from a trade-off between being detected by conspecifics and concealment from potential predators [43].

### Habitat suitability models of Little and Great Bustards

MaxEnt was selected for modelling the spatial distribution of each study species since it is a presence-only approach. This is a machine-learning method based on the principle of maximum entropy [44] that has been employed widely in many ecological studies (for further details see [41], [45]). MaxEnt models have been proved to yield one of the highest quality predictions among several modelling methods and the best performance at low sample sizes [46]–[48].

The species distribution modelling required two independent set of observations, one for calibrating the model and the other for evaluating model predictions [26]. Models were built separately for each species and study site with datasets from years 2008 and 2011 for Calatrava and Campo Real respectively. The regularization parameters to reduce model over-fitting were selected automatically by the program [41]. Predictive maps of probability of habitat suitability for each species and study site were built from calibration datasets and subsequently transformed to Boolean maps of presence/absence by selecting a threshold. We decided to use the average suitability approach [49], which fixes the threshold at the mean of all predicted cell values from the calibration dataset. This approach was chosen because it does not require true absence data and because of its effectiveness and simplicity [50].

Models were evaluated using 2009 and 2012 datasets respectively for Calatrava and Campo Real. Model evaluation should deal with two aspects, the performance and the significance of the model [26]. First, model performance shows how well or poorly the model classifies presence and absence of the species. Omission error rate (the proportion of presence occurrence records of the evaluation dataset that fall in an area predicted as unsuitable for the species) was used as a measure of model performance, expecting low omission rates for good models [26]. This measure of model performance was selected because it does not need true absence records for its calculation [26]. Second, it is also necessary to assess model significance, ie. whether the model predicts presence occurrence records from the evaluation dataset better than expected under random prediction [26]. Thus, one-tailed binomial tests (one per model) were performed to evaluate whether the proportion of correctly classified occurrences differs from the proportion of area predicted as presence by the model.

### Coexistence and exclusive use areas of Little and Great Bustards

Since we were mainly interested in the delimitation of areas in which both species might coexist, a coexistence map was built in each study site. Coexistence maps were generated by superimposing both the Little and the Great Bustard Boolean maps, generating a new one with four cell types: 1) cells predicting presence of both species, 2) cells predicting only Little Bustard presence, 3) cells predicting only Great Bustard presence and 4) cells predicting absence of both species. The surface and density of each species for coexistence, exclusive use and absence areas were calculated in each study site. In addition, means of each land use cover in coexistence and exclusive use areas were calculated in order to describe the land use composition of each area type. Finally, we evaluated habitat suitability differences between coexistence and each species exclusive use areas. In order to eliminate the spatial trends of the data we used a third order polynomial regression with the spatial coordinates [51]. Residuals of the regression were analysed by a Student t test to determine whether probabilities of habitat suitability differ between these area types for both species.

Environmental predictors were generated using ArcGis v9.3 program [52]. TPI was built by the extension “Topographic Position Index (TPI) v 1.2” [53] and MaxEnt modelling was performed by the package “dismo” [54] for the R software v2.14 [55].

## Results

Campo Real presented densities of 4.02 Little Bustards and 5.6 Great Bustards/km^2^ in 2011, higher than the 2.48 Little Bustards and 1.9 Great Bustards/km^2^ found in Calatrava 2008.

Habitat suitability models of Little and Great Bustards predicted the distribution of evaluation points accurately and better than random for the two study sites (Table 1). Little Bustard models predicted a greater extension of presence area than Great Bustard models for both study sites. Little Bustard model in Campo Real showed the highest predicted presence area as well as the lowest omission error rate, predicting correctly almost all the evaluation data set (Table 1).

**Table 1:** Percentage of predicted presence area of Little and Great Bustards in Campo Real 2012 and Calatrava 2009 (corresponding with the evaluation datasets).

	Campo Real		Calatrava	
	Little Bustard	Great Bustard	Little Bustard	Great Bustard
Predicted area (%)	72.07	49.73	58.50	54.55
Omission error rate	0.09	0.33	0.21	0.11
P	0.003	0.0375	<0.001	<0.001

Omission error rates (proportion of presence occurrence records of the evaluation dataset that fall in an area predicted as unsuitable for the species) and p-values of one-tailed binomial test for evaluating model performance and significance respectively are provided.

**Figure 2:**
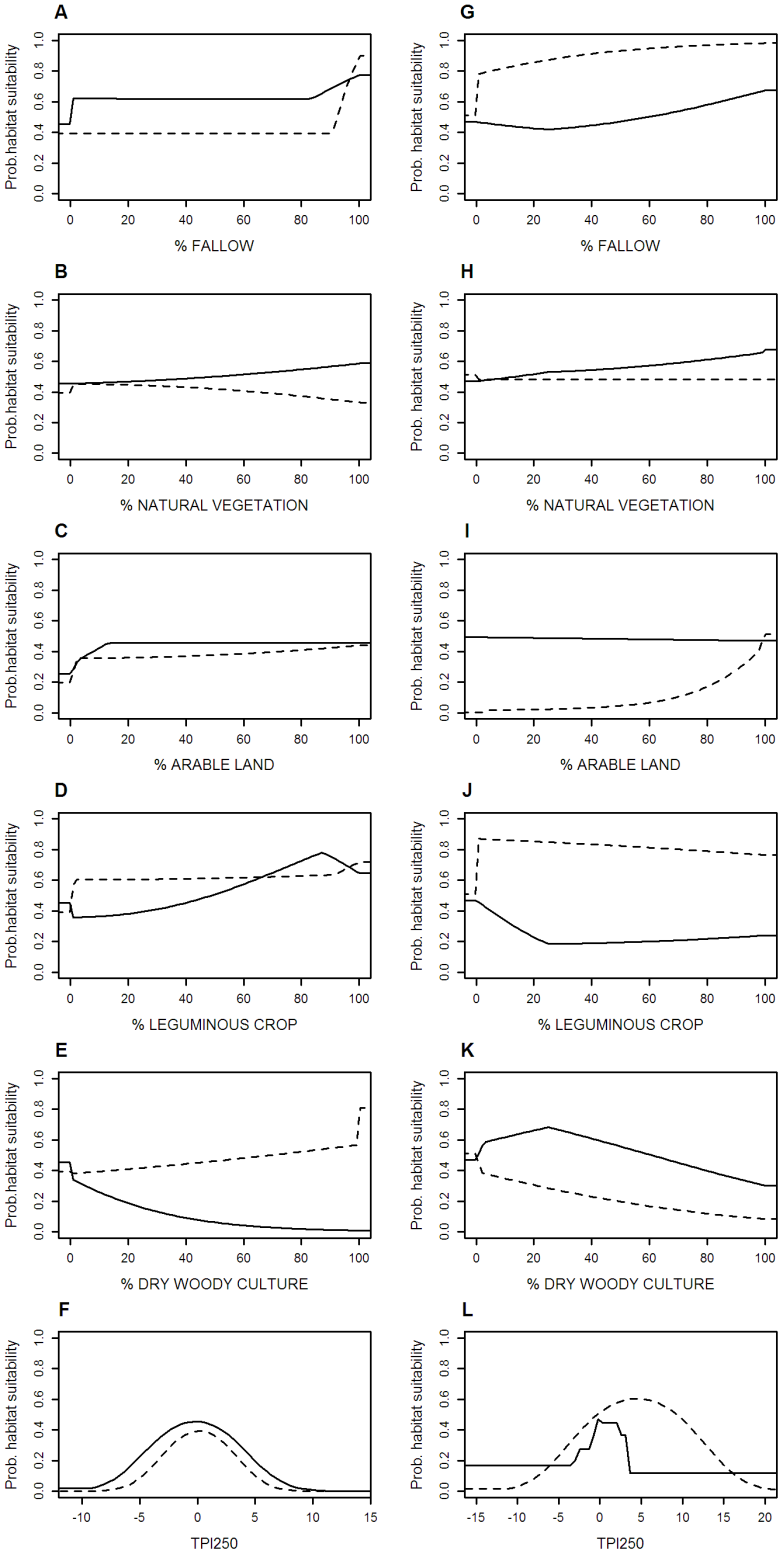
Probabilities of habitat suitability for the environmental predictors. Maxent response curves representing the probability of habitat suitability for each environmental predictor (percentage of land uses and Topographic position index at 250 m resolution, TPI250) for the study species in Campo Real (A–F) and Calatrava (G–L). Solid lines correspond to Little Bustard response curves while broken lines correspond to Great Bustard response curves.

Models for Little Bustard were influenced mainly by the presence of dry woody cultures and fallows as shown by their contribution percentages (ie. the relative contribution of each variable to the model. Table 2). The response was positively related to fallow cover while the cover of dry woody cultures was negatively related with the predicted probabilities of habitat suitability in both study sites (Fig. 2). The cover of leguminous crops was also an important variable, positively related with the Little Bustard predicted distribution in Campo Real (Fig. 2). TPI was one of the most relevant environmental predictors in Calatrava, with highest predictive power at values around 0, indicating a preference for flat zones (Fig. 2).

**Table 2:** Contribution percentages of each environmental predictor (percentage of each land use type, and Topographic position index at 250 m resolution) to each species and study site models yielded by MaxEnt.

	Campo Real		Calatrava	
	Little Bustard	Great Bustard	Little Bustard	Great Bustard
Fallows	20.60	37.41	43.16	5.59
Natural Vegetation	0.56	5.98	4.49	1.33
Arable	11.39	16.32	2.10	77.82
Dry woody cultures	44.94	5.45	20.24	10.36
Leguminous crops	19.74	28.08	0.50	1.10
TPI250	2.77	6.78	29.49	3.81

Models were built using Little and Great Bustard observations from 2011 for Campo Real and 2008 for Calatrava.

Differences between study sites were greater in Great Bustard models. Arable land appeared as one of most relevant predictors, especially in Calatrava's model (Table 2). Campo Real's model was highly influenced also by the presence of fallows and leguminous crops, both showing a positive relationship with the predicted probability of habitat suitability (Fig. 2).

**Figure 3:**
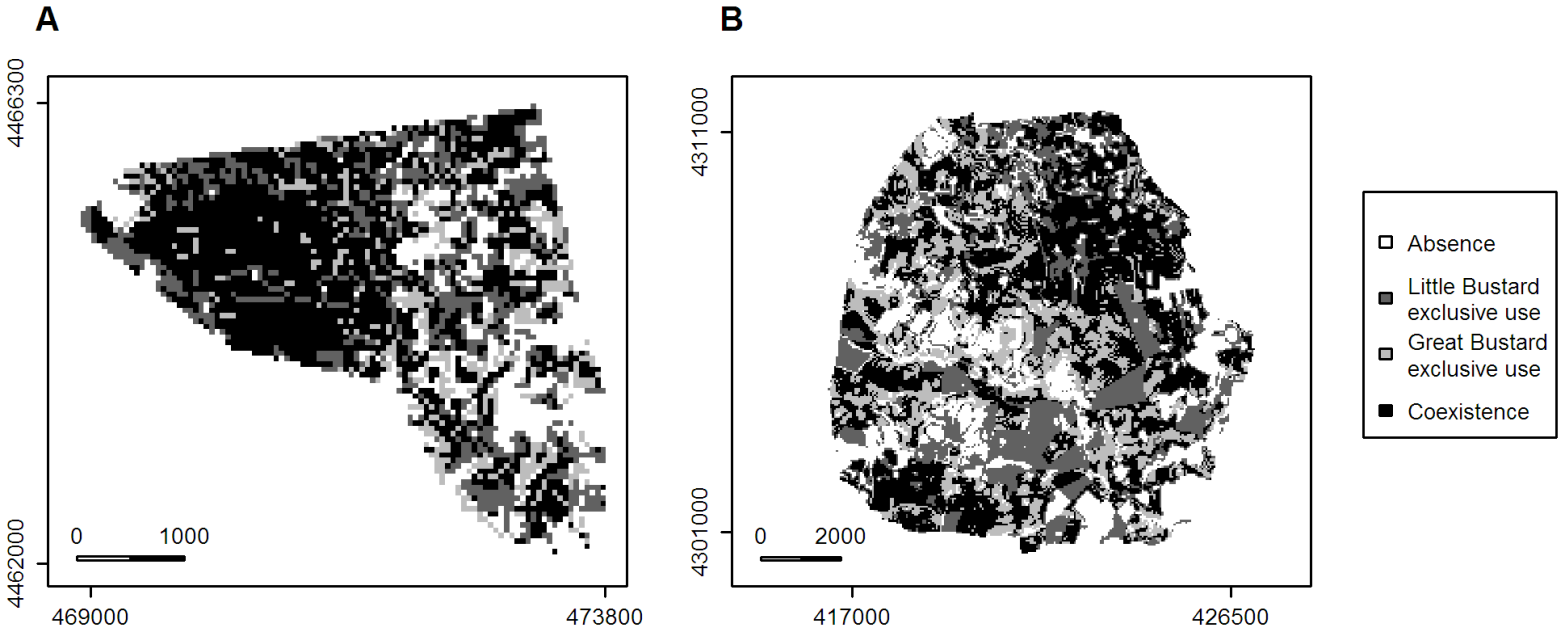
Coexistence maps of Little and Great Bustards. Maps of Little Bustard and Great Bustard coexistence for 2011 in Campo Real (A) and 2008 in Calatrava (B), showing also areas of exclusive use and areas in which both species were predicted to be absent. The scale bar is given in meters.

In Campo Real, 45.33% of the surface corresponded to the coexistence area (Fig. 3). The Little Bustard exclusive use area presented a cover value of 20.78%, whereas the Great Bustard exclusive area reached a lower cover of 12.62%. In Calatrava, the predicted coexistence area accounted for the 36.15% of the surface (Fig. 3), lower than the value found in Campo Real. The area predicted as exclusively used by the Little Bustard in Calatrava reached 22.38% cover, while the predicted Great Bustard exclusive area was 20.80%.

**Figure 4:**
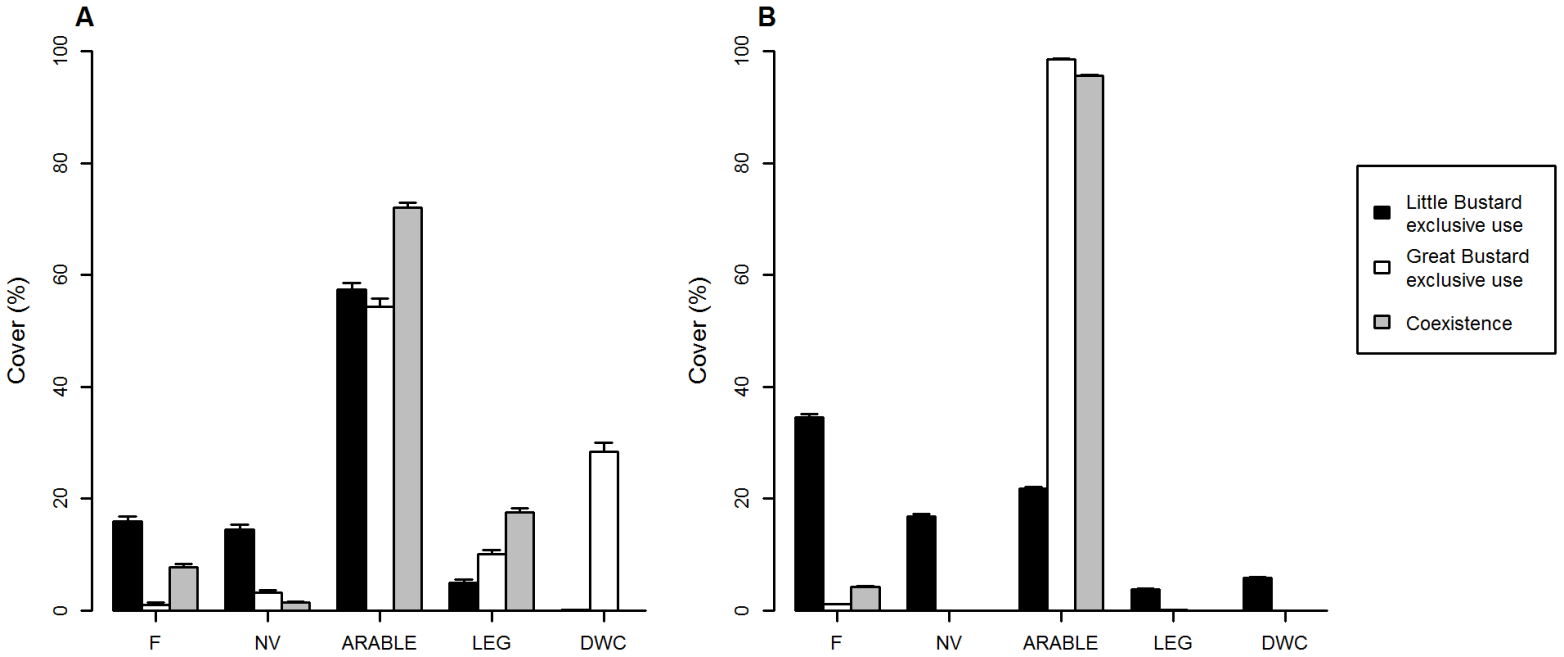
Land use cover in each area type. Mean and standard error of land use cover in the predicted Little and Great Bustard exclusive use and coexistence areas for 2011 in Campo Real (A) and 2008 in Calatrava (B) (F: short term fallows, NV: natural vegetation encompassing long term fallows and low height scrubs; Arable: cereal fields and ploughed lands; LEG: leguminous crops; DWC: dry woody cultures).

In Campo Real, the density of Little Bustards in the predicted coexistence area was slightly higher than in the exclusive use area (Table 3). The same pattern was found for Great Bustards in Calatrava site. However, densities in coexistence area were lower than in exclusive use area in the case of Little Bustard in Calatrava and Great Bustard in Campo Real (Table 3). Regarding land use composition, Little Bustard exclusive use areas showed a higher cover of fallows and natural vegetation than Great Bustard exclusive use and coexistence areas in both study sites (Fig. 4). Little Bustard exclusive use area showed a lower value of arable surface in Calatrava than in Campo Real. In addition, this value was also lower than the cover of Great Bustard exclusive use and coexistence areas in both study sites (Fig. 4).

**Table 3:** Densities of Little (males/km^2^) and Great Bustards (individuals/km^2^) in the different area types generated by superimposing the predicted presence maps of Little and Great Bustards for 2011 in Campo Real and 2008 in Calatrava.

	Campo Real		Calatrava	
	Little Bustard	Great Bustard	Little Bustard	Great Bustard
Absence area	2.46	0.41	0.81	0.05
Little Bustard exclusive area	5.04	0.84	4.71	0
Great Bustard exclusive area	0.69	5.54	1.23	1.01
Coexistence area	5.20	3.66	2.52	1.35

**Figure 5:**
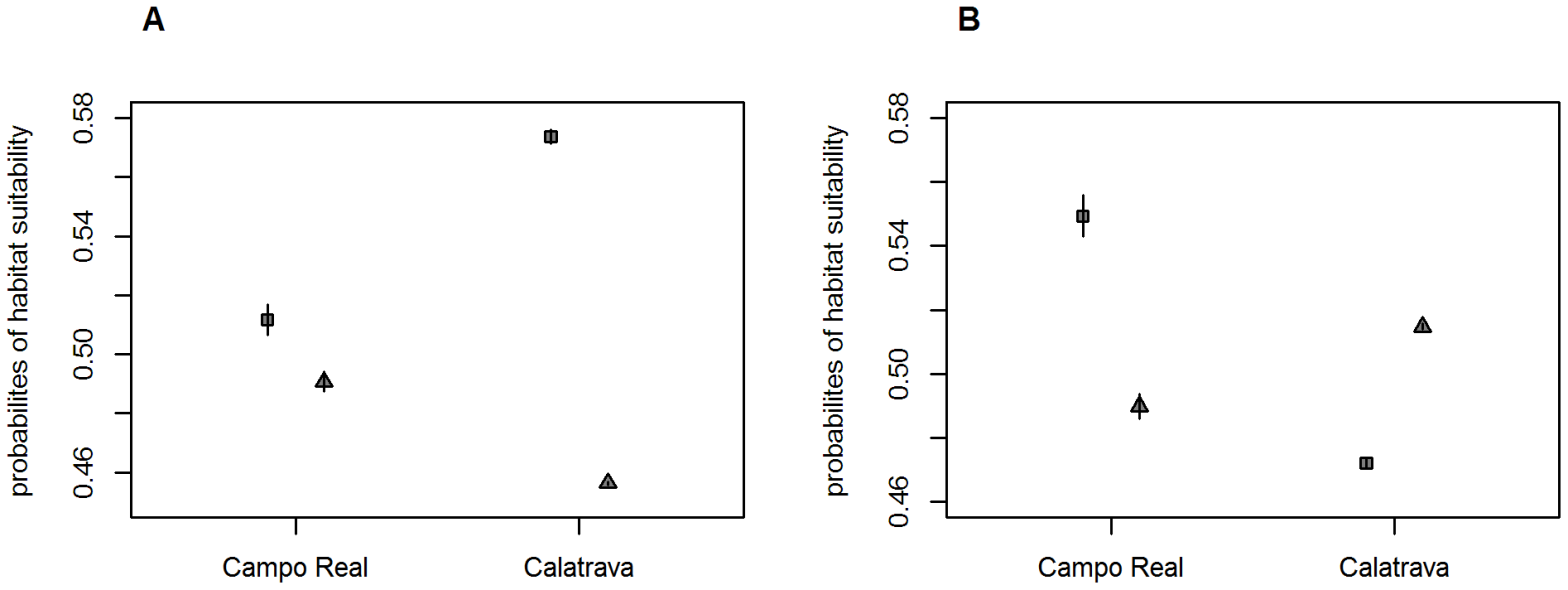
Probabilities of habitat suitability of coexistence and exclusive use areas. Mean and 95% confidence interval of probabilities of habitat suitability in coexistence and exclusive use areas for the Little (A) and Great Bustards (B) in Campo Real 2011 and Calatrava 2008. Student t tests were performed with the residuals of the polynomial regression although original probabilities are shown for the sake of interpretation. Probability means of coexistence areas are represented as triangles and probability means of exclusive use areas are represented as squares.

The residuals of the polynomial regression were significantly different between coexistence and exclusive use areas for both species in both study sites. The Little Bustard showed higher probabilities of habitat suitability in areas where only this species was predicted as present than in areas in which it might coexist with the Great Bustard (Campo Real: t_0.05;1868.391_ = 12.047, p<0.001; Calatrava: t_0.05; 9703.717_ = 98.200, p<0.001, Fig. 5). The same pattern was found for Great Bustards in Campo Real (t_0.05; 1150.884_ = 21.817, p<0.001), although this species showed higher probabilities of habitat suitability in coexistence areas than in areas of exclusive use in Calatrava (t_0.05; 13177.676_ = −27.053, p<0.001, Fig. 5).

## Discussion

The models yielded by MaxEnt for two endangered bird species linked to pseudo-steppe landscapes, the Little and the Great Bustards, were able to predict suitable areas accurately. It is important to note that Little Bustard results correspond only to males and conclusions may not apply to females which might show a different habitat selection pattern. Our results showed that models are not only species-specific but also context-dependent. Little Bustard presence areas seem to be the result of a more complex combination of different substrate types while the Great Bustard shows a higher dependence on arable fields. Coexistence areas are also context-dependent at local scale and tend to harbour less suitable habitat than areas of exclusive use. The results found in this study have implications for conservation and management strategies.

The Little and the Great Bustards have been the object of many habitat selection studies due to their interest as lekking species and their worrying conservation status caused by changes in agricultural practices during the last decades. Our models showed that both species benefit from the presence of short term fallows in accordance with previous studies [32], [34], [56]. Little Bustard males' preference for short term fallows as habitats that ensure conspicuousness for sexual displaying and food supply [32], [56], is reflected in our models by their high contribution percentages. In the case of Great Bustard, the importance of fallow cover in explaining the distribution pattern seems particularly context-dependent. In Campo Real, fallows appear as a relevant substrate type for Great Bustard while the effect on its distribution is minimal in Calatrava. Leguminous crops play also an important role for both species when they are present in the landscape. In the case of Little Bustard, leguminous crops reach a similar importance in the model as fallow lands in Campo Real, but remain as a minor variable in Calatrava, where the presence of this substrate is clearly marginal.

However, these species differ in their responses to other landscape variables, indicating some level of niche segregation at local scale. For instance, the relevance of arable lands is clearly different between species, being the cover of this land use more important for the Great Bustard. The presence of dry woody cultures plays a minor role in the distribution pattern of the Great Bustard but not for the Little Bustard, which avoids vineyards and olive groves in accordance with previous studies [32], [57]. Finally, the importance of topography varies between species and study sites. The Little Bustard shows in both study sites the same preference for flat zones where they are visible to other individuals during the sexual display season. However, the relevance of flat zones changes with the study site, being especially high in Calatrava, which might be due to its higher variability in topography. In the case of the Great Bustard, its distribution pattern is hardly affected by topography, while land use variables acquire a major role in determining the species' distribution in both study sites. The differences found between study sites might be indicating that habitat selection depends on the particular landscape composition. This is especially noteworthy for the Great Bustard, which may be explained by its greater niche width [58]. Nevertheless, results might also be influenced by the SDMs' dependency on the environmental context, since the model calibration process depends on the particular combination of variables that occurs in each study site [26]. Although the spatial scale selected may influence observed response patterns, this seems to occur only at high cover values of some land uses (Fig. 2). In any case, results are consistent with the existing habitat selection knowledge for the species, as pointed out previously.

Our results show that concentrating conservation efforts on preserving the habitats most preferred by one species at local scale may be detrimental for the other given their different requirements, leaving habitats relevant to that species without protection. Therefore, a multi-species approach may help prioritize conservation efforts on coexistence areas. Our study shows that coexistence and exclusive use areas of Little and Great Bustards differ in their habitat features, which may also vary in relation to the local environmental context. The area predicted as suitable for the coexistence of these species is greater than the surface of each species exclusive use in both study sites. However, different situations emerge when looking at probabilities of habitat suitability and actual densities. In two cases, Little Bustard in Calatrava and Great Bustard in Campo Real, the corresponding exclusive use area harbours better habitat conditions for the target species and also higher density. Thus, the coexistence area might correspond to suboptimal zones for the species. However, we cannot disentangle whether the low probabilities of habitat suitability predicted for coexistence areas are due to poor habitat quality or to the avoidance of heterospecifics since both factors can affect distribution patterns [11]. The other two cases (Little Bustard in Campo Real and Great Bustard in Calatrava) present similar densities but different habitat suitability for each area type. The exclusive Little Bustard area in Campo Real shows higher habitat suitability than the coexistence area. It seems that Little Bustards might occupy less suitable areas in the absence of enough space or good quality habitats. However, the pattern for Great Bustards in Calatrava is the opposite, with higher probabilities of habitat suitability in coexistence areas. Therefore, the coexistence area in Calatrava seems to reflect Great Bustard habitat preferences whereas Little Bustards concentrate mainly in their exclusive use area. Low density might allow Little Bustards to occupy their most preferred habitat features without using areas suitable for the Great Bustard. It is noteworthy that each species presents lower densities in the absence and exclusive use areas of the other species, a fact that might support the hypothesis of segregation between these two steppe-birds. Consequently, by prioritizing the preservation of coexistence areas, we may be protecting low quality habitats that are being used by the two (or more) species because higher quality exclusive areas are scarce, thus preventing natural between-species avoidance.

Some interesting conservation consequences arise from this study. Both species seem to benefit from high percentage of short-term fallows and leguminous crops at landscape scale, so that promoting the application of agri-environmental schemes that favour the concentration of these habitats in small areas in the landscape is desirable. In this context, Concepción and Díaz [59] emphasized that the effects of agri-environmental schemes are limited by their application at field level, and plans designed at landscape level are needed to maintain the mosaic structure of this extensive cereal croplands. For instance, the traditional two-year rotation system known as Iberian dry-farming would benefit species linked to extensive cereal croplands since it maintains a complex and dynamic structure of different and complementary land uses [60]. However, their different habitat preferences constrain the potential delimitation of coexistence areas encompassing high quality habitats at local scale. In order to meet species' spatial requirements, protected areas for these (and probably other) steppe birds should cover territories large enough to allow their coexistence by the selection of their preferred areas, or their tendency to segregate in space. Therefore, the role played by biotic interactions in a community should be considered when designing conservation strategies at least at local scale. Finally, the context-dependence of habitat selection in these species advices designing conservation measures for particular landscape situations.

Spatial distribution modelling is a useful tool for species conservation since it can integrate behavioural traits and landscape measurements and helps identifying general responses to environmental variables. In addition, it allows the extrapolation of results to other regions in order to preserve non-occupied areas of suitable habitat that could be potentially colonized at long term [61]. This is important even in the case of the Great Bustard whose strong breeding philopatry constrains the colonization of unoccupied areas [62].

## Conclusions

The identification of coexistence areas of two farmland birds at local scale described in this study provides insightful results that might apply in other cases. Concentrating efforts on one umbrella species may be hazardous if that species does not adequately reflect the ecological requirements of sympatric heterospecifics. Hence, a multi-species approach may be more adequate, and the identification of coexistence areas may provide an idea of the spatial requirements of a particular assemblage. However, when coexistence areas correspond to suboptimal habitats for species that would be otherwise segregated due to their different ecological requirements, focusing efforts on these areas may be misleading at local scale. Moreover, the influence of the local environmental context in determining coexistence areas is not detected at broader scales, at which species sharing requirements overlap in their distribution ranges. Finally, integrating information of species distribution models built at local scale might lead to a better understanding of general patterns at broader scales [7].
